# Role of CD138^+^ Plasma Cells and Natural Killer Cells in Couple Infertility: A Review

**DOI:** 10.3390/biomedicines13092248

**Published:** 2025-09-12

**Authors:** Alessandro Messina, Alessandro Libretti, Ilaria Giovannini, Livio Leo, Valentino Remorgida, Bianca Masturzo, Raffaele Tinelli

**Affiliations:** 1 Department of Obstetrics and Gynecology, University Hospital “Degli Infermi”, 13875 Ponderano, Italy; alessandro.messina@aslbi.piemonte.it (A.M.); ilaria.giovannini27@gmail.com (I.G.); bianca.masturzo@aslbi.piemonte.it (B.M.); 2 Department of Gynaecology and Obstetrics, University Hospital Maggiore Della Carità, Corso Mazzini, 18, 28100 Novara, Italy; valentino.remorgida@uniupo.it; 3 Department of Surgical Science, Hopital Beauregard, AUSL Valle d’Aosta, 11100 Aosta, Italy; lleo@ausl.vda.it; 4 Department of Obstetrics and Gynecology, “Valle d’Itria” Hospital, 74015 Martina Franca, Italy

**Keywords:** infertility, chronic endometritis, CD138^+^ plasma cells, natural killer cells, implantation failure

## Abstract

Couple infertility is a multifactorial condition that involves, among other factors, immunological alterations of the endometrium. CD138^+^ plasma cells and uterine Natural Killer (NK) cells have been associated with implantation failures and recurrent pregnancy loss, although the literature presents considerable heterogeneity in findings. This narrative review aims to synthesize current evidence on the role of these two immunological biomarkers in the pathophysiology of infertility. According to the current literature, the presence of CD138^+^ plasma cells was strongly associated with chronic endometritis and adverse reproductive outcomes, with improvement following targeted antibiotic therapy. NK cells, particularly in their hyperactive peripheral form, were elevated in infertile women, although data on uterine NK cells remain controversial. Recent studies suggest a possible interaction between chronic inflammation and NK dysfunction. A combined evaluation of CD138^+^ and NK cells may represent an integrated diagnostic approach in idiopathic infertility. Prospective studies are needed to standardize cut-off values and validate these markers in clinical practice.

## 1. Introduction

Infertility is a clinical condition defined as the inability to achieve pregnancy after at least 12 months of regular, unprotected sexual intercourse [1]. According to the World Health Organization (WHO), it affects approximately 10–15% of couples of reproductive ages, with a significant impact on quality of life, psychological well-being, and social and family dynamics [2,3]. The causes of infertility may be male, female, combined, or idiopathic, with the latter accounting for up to 30% of cases [4]. Idiopathic infertility represents a relevant clinical challenge, suggesting the existence of pathophysiological mechanisms that are not yet fully understood or are underdiagnosed [5].

In recent years, growing attention to the role of the immune system in regulating reproductive function has opened new perspectives in understanding the causes of unexplained infertility [6]. Specifically, the importance of the uterine immunological environment has emerged, since the endometrium is not merely a receptive tissue but a dynamic microenvironment modulated by complex interactions among stromal, epithelial, and immune cells [7]. These include dendritic cells, T lymphocytes, Natural Killer (NK) cells, and plasma cells, each with specific roles in regulating maternal–fetal immune tolerance, embryo implantation, and placental development [8,9].

Two immune cell populations have received significant attention for their potential impact on reproductive outcomes: CD138^+^ plasma cells and uterine Natural Killer (uNK) cells. CD138^+^ plasma cells, identified via immunohistochemistry as markers of the syndecan-1 antigen, are the main diagnostic component of chronic endometritis (CE), a subclinical inflammatory condition that is often asymptomatic but strongly associated with implantation failure, recurrent pregnancy loss (RPL), and unsuccessful assisted reproductive technology (ART) outcomes [10,11]. Chronic endometritis may alter endometrial gene expression, compromising the implantation window and embryo acceptance by the uterus [12].

Among the immune cell populations present in the endometrium, two have emerged as particularly relevant in the context of reproductive failure: CD138^+^ plasma cells and uterine Natural Killer (uNK) cells [7].

CD138^+^ plasma cells, identified by the syndecan-1 surface marker (CD138) through immunohistochemistry, are terminally differentiated B cells involved in chronic inflammation. In the endometrial environment, their accumulation is the hallmark of chronic endometritis (CE)—a low-grade, often asymptomatic inflammatory condition that alters endometrial receptivity [3,9]. Mechanistically, CE impairs the uterine environment by disrupting cytokine networks, downregulating implantation-related genes (e.g., LIF, HOXA10), and promoting stromal fibrosis [5]. These alterations can interfere with embryo implantation and placental development, contributing to recurrent implantation failure (RIF), recurrent miscarriage, and unexplained infertility [11]. The presence of CD138^+^ plasma cells in endometrial tissue has thus become a central diagnostic criterion for CE, with evidence suggesting improved pregnancy outcomes following targeted antibiotic treatment [4,7,11,12,13,14].

In parallel, uterine NK (uNK) cells—distinct from peripheral NK cells—constitute the most abundant lymphocyte population in the endometrium during the secretory phase. They exhibit a unique CD56^bright^ CD16^−^ phenotype and possess limited cytotoxicity under physiological conditions [10,15]. Their main functions include secreting angiogenic factors (e.g., VEGF), supporting decidualization, and modulating maternal–fetal immune tolerance. However, dysregulation of uNK cells—either in number, receptor profile (e.g., altered KIR-HLA-C interactions), or cytokine secretion—has been implicated in impaired implantation and early pregnancy loss. Abnormal activation or overexpression of cytotoxic mediators such as perforin and granzyme B may create a hostile endometrial environment, leading to embryo rejection [6,14,16,17].

While both cell types independently affect endometrial receptivity, emerging evidence suggests they may also interact within the inflammatory milieu. For example, chronic endometritis marked by CD138^+^ cells may indirectly modulate NK cell activity, further compounding implantation failure. Understanding the mechanistic interplay between these two immune populations is essential for advancing personalized diagnostic and therapeutic strategies in infertility [9].

Despite increasing evidence of immune involvement in fertility, there is still a lack of an updated synthesis that integrates the combined roles of CD138^+^ plasma cells and NK cells in the diagnosis and treatment of infertility [18]. A deeper understanding of endometrial immunodynamics could clarify the pathophysiological basis of idiopathic infertility and guide the development of personalized diagnostic and therapeutic strategies, including the use of antibiotics, corticosteroids, immunomodulators, or cellular therapies [19].

Considering these, the aim of this narrative review is to critically analyze the current scientific literature regarding the role of CD138^+^ plasma cells and Natural Killer cells in couple infertility, with particular attention to clinical, diagnostic, and therapeutic implications. Through an analysis of the published studies, we aim to understand whether these immune markers may serve as reliable biomarkers and therapeutic targets to improve reproductive outcomes in infertile couples [20].

Historically, the hypothesis of immune dysregulation in infertility stems from analogies with transplant immunology, where maternal immune tolerance toward the embryo resembles graft acceptance. Early histological findings of lymphocytic infiltrates in women with repeated implantation failure initiated a wave of investigations into endometrial immunobiology [8,12,19].

Moreover, recent studies highlight that the local immune milieu is not static but dynamically modulated throughout the menstrual cycle and embryo implantation window. The cyclical recruitment and activation of immune cells—especially NK cells and plasma cells—are tightly regulated by sex hormones. Disruption in this balance may lead to pathological conditions such as chronic endometritis or aberrant uNK cell activation.

Technological advancements in endometrial sampling, immunohistochemistry, and flow cytometry have improved our ability to profile these immune populations. Nonetheless, the lack of uniform diagnostic thresholds and the heterogeneity of clinical practices continue to hinder widespread implementation of immune testing in infertility workups [8,12,19].

## 2. Materials and Methods

### 2.1. Study Design

Although a systematic approach has been adopted to retrieve the study to analyze, the formula of a narrative review has been chosen for this study, due to the nature of the topic. This narrative review was conducted anyway in accordance with the PRISMA (Preferred Reporting Items for Systematic Reviews and Meta-Analyses) guidelines [21] to ensure a rigorous and transparent approach to the selection, evaluation, and synthesis of included studies. The objective of the review was to summarize and critically assess the available literature regarding the role of CD138^+^ plasma cells and Natural Killer cells in the pathophysiology of couple infertility.

### 2.2. Sources and Search Strategy

A literature search was performed using the PubMed/MEDLINE database, covering publications up to June 2025. The search strategy combined Medical Subject Headings (MeSH) with free-text keywords in English to capture the widest range of relevant publications [22]. The following search string was used:

- (“Infertility”[MeSH Terms] OR “Infertility, Female”[MeSH Terms] OR infertility[Title/Abstract])
- AND (“CD138”[Title/Abstract] OR “syndecan-1”[Title/Abstract] OR “plasma cells”[MeSH Terms])
- AND (“Natural Killer Cells”[MeSH Terms] OR “NK cells”[Title/Abstract])
- AND (“Endometrium”[MeSH Terms] OR endometrium[Title/Abstract] OR uterus[Title/Abstract])
- The search was further refined to include only:
- Articles published in English.
- Full-text available studies.
- Studies conducted on human subjects.

Additionally, the references of the included articles were manually screened to identify further relevant sources not captured in the initial search [23].

### 2.3. Inclusion and Exclusion Criteria

Inclusion criteria:

- Observational studies (cohort, case–control, cross-sectional) and clinical trials (randomized or not) that evaluated the role of CD138^+^ plasma cells and/or NK cells in patients with infertility, recurrent implantation failure (RIF), or recurrent spontaneous abortion (RSA) [24].
- Studies that included either quantitative or qualitative assessment of CD138^+^ plasma cells (via immunohistochemistry) or NK cells (either peripheral or uterine, using flow cytometry, immunohistochemistry, or endometrial biopsy).
- Comparative studies with fertile control groups or pre-/post-treatment designs.

Exclusion criteria:

- Preclinical studies on animal models or in vitro cell lines.
- Abstracts without full-text availability.
- Case reports, editorials, letters to the editor, and narrative reviews (except when used as secondary references).
- Studies not directly related to infertility (e.g., oncology, systemic autoimmunity)

### 2.4. Study Selection Process

**Figure 1:**
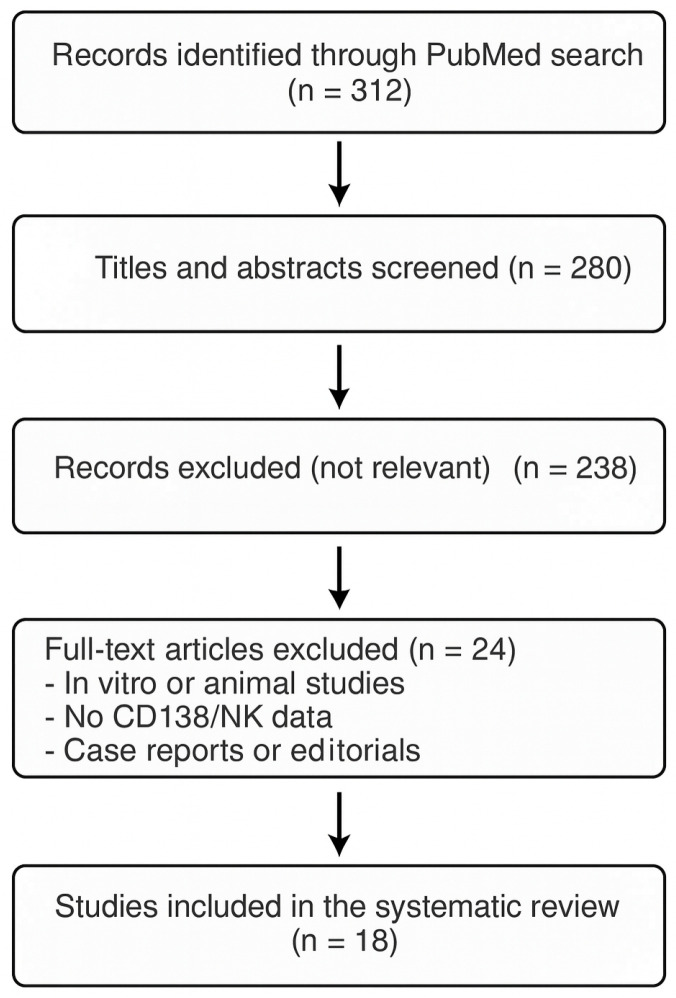
PRISMA flowchart.

Two independent reviewers (R1 and R2) screened the titles and abstracts of all identified articles. Studies considered potentially relevant were retrieved in full text and assessed for eligibility. Disagreements between reviewers were resolved by discussion and, if needed, consultation with a third reviewer (R3). The entire selection process was summarized in a PRISMA flow diagram (Figure 1), illustrating the number of records identified, screened, excluded, and ultimately included in the review [25]. Figure 1 represents the PRISMA flowchart.

## 3. Results

A total of 312 articles were initially identified through the search of the PubMed database. After removing duplicates and screening titles and abstracts, 42 articles were selected for full-text assessment. Of these, 18 met all inclusion criteria and were included in this narrative review. The studies span the period from 2012 to 2024 and include both observational studies and meta-analyses.

### 3.1. CD138^+^ Plasma Cells and Chronic Endometritis: Reproductive Implications

Several studies have demonstrated a significant association between the presence of CD138^+^ plasma cells in the endometrium and infertility [10,11,24,26,27,28]. In particular, chronic endometritis (CE), diagnosed via immunohistochemistry for CD138, has frequently been observed in women with recurrent implantation failure (RIF) and recurrent spontaneous abortion (RSA) [11,25,26,27,28].

A retrospective cohort study by Li et al. (2024) involving 664 women undergoing IVF showed that the presence of ≥3 CD138^+^ lesions (with a threshold of ≥5 cells per high-power field) was significantly associated with reduced clinical pregnancy and live birth rates [29]. Endometrial biopsies were performed during the proliferative phase, highlighting that the diagnostic sensitivity for CE may vary depending on the phase of the menstrual cycle [30]. Previous studies had already suggested that the follicular phase is optimal for detecting plasma cells, as the luteal phase may show physiologic inflammatory infiltration, leading to false positives [31].

Other studies have reported improved reproductive outcomes following targeted antibiotic treatment of CE, with subsequent immunohistochemical confirmation of CD138^+^ clearance [12,32]. These findings support the hypothesis that CE is a reversible condition with significant clinical implications [32].

Moreover, longitudinal studies measuring NK cell profiles before and after immunomodulatory therapy suggest that dynamic changes—rather than static levels—may better predict clinical outcomes. For instance, normalization of NK cytotoxic markers (e.g., CD69, NKG2D) after corticosteroid therapy correlated with increased implantation rates in selected patient groups [12,31,32].

### 3.2. Natural Killer Cells and Fertility: A Dual Role of Support and Cytotoxicity

NK cells, especially uterine NK (uNK) cells (CD56^bright^ CD16^−^), are the dominant immune population in the endometrium during the secretory phase. They contribute to angiogenesis, implantation support, and fetal immune tolerance [13]. However, elevated numbers or altered cytotoxic activity of these cells may be detrimental [14,16].

A meta-analysis by Tang et al. (2013), including 22 observational studies, reported that infertile women had significantly higher absolute numbers of peripheral NK cells compared to fertile controls [16]. However, findings related to uNK cells were more conflicting: some studies reported no significant differences in uNK percentages between fertile and infertile women, suggesting that not only quantity but also functional profiles (e.g., granzyme B, perforin, KIR expression) may influence outcomes [17,33].

There was notable heterogeneity in the methods used to quantify NK cells, with some studies using peripheral blood flow cytometry and others using endometrial biopsy and immunohistochemistry [17]. The lack of methodological standardization is a recurrent limitation in current research [17].

Preliminary clinical trials suggest that immunomodulatory treatments (e.g., corticosteroids, intralipids, intravenous immunoglobulins) may reduce NK cytotoxicity and improve implantation rates, although the efficacy of such approaches remains debated [34].

Interestingly, some studies have observed a paradoxical association between normal uNK cell counts and poor reproductive outcomes, leading to hypotheses about functional exhaustion or inappropriate receptor–ligand interactions. Specific killer immunoglobulin-like receptor (KIR) genotypes and their interactions with HLA-C ligands on trophoblasts may determine the immunological “compatibility” between mother and embryo, potentially affecting implantation success [16,19,34].

### 3.3. Immune Interaction: CD138^+^ and NK Cells as Combined Biomarkers

Few studies have simultaneously assessed the presence of CD138^+^ plasma cells and NK cells in the endometrium. However, emerging hypotheses suggest that the interaction between these two cell populations may reflect a broader immune imbalance, in which latent chronic inflammation (mediated by CD138^+^) is coupled with dysregulated innate immune activity (mediated by NK cells) [15].

A prospective study by Kitaya et al. (2036) analyzed endometrial biopsies in women with RIF and unexplained infertility, evaluating combined expression of CD138, CD56, and BCL-6. The concomitant presence of these three markers was associated with significantly lower clinical pregnancy rates after embryo transfer, indicating that an integrated immunological evaluation of the endometrium may have predictive value [20,35].

Moreover, chronic endometrial inflammation may alter NK cell distribution and function, creating a hostile endometrial microenvironment for embryo implantation [13,19]. Thus, the combined use of CD138^+^ and NK markers may provide a more accurate diagnostic approach than evaluating each parameter in isolation [35,36].

Additional insight comes from gene expression studies showing that pro-inflammatory transcription factors (e.g., NF-κB) are upregulated in endometria with dual positivity for CD138^+^ and NK markers. This suggests that chronic inflammation may create a pro-rejection environment unfavorable to embryo implantation [13,19].

These findings also raise the possibility of therapeutic synergy. Some pilot trials have explored sequential therapy with antibiotics followed by corticosteroids in women with mixed CE and uNK abnormalities, reporting modest improvements in pregnancy rates. However, robust data from randomized controlled trials are lacking [13,19].

## 4. Discussion

This narrative review highlights the growing interest in the use of endometrial immunological biomarkers, particularly CD138^+^ plasma cells and Natural Killer cells, to understand the pathogenesis of couple infertility [8,13,20]. Available data suggest that both cell types can negatively influence the uterine environment and impair embryo implantation, especially in cases of unexplained infertility, RIF, and RSA [10,14,24]. However, the strength of the evidence varies, and substantial gaps remain regarding the standardization of definitions, diagnostic methods, and treatment criteria [17,31].

### 4.1. Role of CD138^+^ Plasma Cells in Chronic Endometritis

The presence of CD138^+^ plasma cells is widely recognized as a reliable histological marker of CE, a clinically relevant condition that is often asymptomatic [10,24]. Studies, particularly that of Li et al. (2024) [29], have shown that CE is associated with reduced clinical pregnancy and live birth rates, and that targeted antibiotic therapy can significantly improve reproductive outcomes [12,29,32]. However, there is still no universal standard for diagnosing CE: cutoff values for plasma cell counts vary (≥1 to ≥5 per HPF), as does the timing of the biopsy within the menstrual cycle [30,31].

The lack of standardized diagnostic protocols may partly explain the heterogeneity of study findings. In particular, the luteal phase may physiologically present transient lymphoplasmacytic infiltration, increasing the risk of false positives [31]. A consensus-based diagnostic definition supported by international guidelines is needed to unify CE criteria in both clinical and research settings [27].

Emerging studies are also investigating how CE may influence endometrial receptivity at the transcriptomic level. Downregulation of genes associated with implantation, such as LIF and HOXA10, has been observed in CE-positive samples, suggesting a mechanistic link between inflammation and endometrial dysfunction. These molecular changes may not be reversed immediately with antibiotic treatment, highlighting the importance of post-treatment reevaluation [30,31].

In addition, some data suggest a potential link between CE and dysregulation of the endometrial microbiome. It remains unclear whether microbial dysbiosis drives the inflammatory process or is a consequence of immune dysfunction. This opens future avenues for investigating the use of probiotics or microbiota-targeted therapies in infertility management [30,31].

Recent transcriptomic analyses of endometrial tissue with histologically confirmed CE have revealed downregulation of genes critical for implantation, such as *LIF*, *HOXA10*, and *IGFBP1*. Concurrently, there is upregulation of pro-inflammatory pathways, including NF-κB signaling and chemokines like *CXCL13* and *CCL20*. Proteomic studies further support these findings, showing increased expression of inflammatory proteins (e.g., TNF-α, IL-1β) and altered extracellular matrix remodeling enzymes. These molecular disruptions contribute to an endometrial microenvironment that is non-receptive and chronically inflamed [30,31].

### 4.2. NK Cells: Ambiguous Regulators of Embryo Implantation

uNK cells are essential components of the receptive endometrium, supporting decidualization, vascular remodeling, and maternal–fetal tolerance [13]. However, excessive uNK presence or pro-inflammatory functional profiles (e.g., increased granzyme B, perforin, or activating KIR receptors) have been linked to implantation failure and recurrent miscarriage [14,33].

Tang et al.’s meta-analysis clarified the role of peripheral NK cells, revealing elevated circulating levels in women with reproductive disorders [16]. The role of uNK cells remains controversial; some studies found no significant differences between fertile and infertile women, emphasizing the need to assess NK functionality, not just quantity [17,33]. Moreover, inconsistent detection methods (flow cytometry vs. immunohistochemistry) and a lack of uniform markers (CD56, CD16, CD69) contribute to the inconsistency of findings [17].

Recent studies have attempted to subclassify uNK cells beyond CD56 and CD16 markers, incorporating CD57, CD94, and KIR profiles to differentiate between regulatory and cytotoxic subsets. Understanding these distinctions may help explain the heterogeneity in clinical outcomes and guide future targeted therapies [14,19,33].

Another important aspect is the potential for immunogenetic mismatches between partners. Certain maternal KIR haplotypes (e.g., AA) in combination with fetal HLA-C2 alleles have been associated with increased risk of preeclampsia and miscarriage, raising questions about immune compatibility as a factor in infertility [14,19,33].

Functional profiling of uNK cells at the transcriptomic level reveals a dual phenotype: while CD56^bright^ uNK cells express angiogenic factors (*VEGF*, *Ang-2*), aberrant populations display increased transcripts for cytotoxic mediators (*PRF1*, *GZMB*, *IFNG*). Studies using single-cell RNA sequencing have identified uNK subpopulations with divergent roles in either supporting implantation or mediating immune rejection. At the proteomic level, excessive activation correlates with secretion of granzyme B, perforin, and IFN-γ, which may disrupt trophoblast invasion and endometrial vascular remodeling [14,19,33].

### 4.3. CD138^+^–NK Interaction: Towards an Integrated Immunological Evaluation

An emerging and particularly interesting area involves the interaction between CD138^+^ plasma cells and NK cells in the endometrium. Although the literature is limited, some studies (e.g., Kitaya et al., 2019 [15]) suggest that chronic inflammation and dysregulated innate immune responses may jointly create an unfavorable uterine environment.

This integrated model supports the idea of “immune-mediated infertility,” in which multiple immune components synergistically (or antagonistically) influence reproductive success [19,35]. This hypothesis opens promising avenues for personalized diagnostics: an immunological panel including CD138, CD56, BCL-6, and NK functional markers may serve as a predictive tool in clinical settings, particularly in ART contexts [35,36]. However, current evidence is still preliminary and requires confirmation in prospective controlled studies [37].

In this context, immunophenotyping of the endometrium using multi-marker panels—integrating CD138, CD56, BCL-6, and Ki-67—could offer a composite risk profile for implantation failure. Some centers have started implementing such panels, albeit with limited consensus on interpretation criteria. Standardization of these approaches may allow for broader clinical utility and comparison across studies [15].

Ultimately, the integration of endometrial immune profiling into ART protocols must be guided by prospective evidence. Randomized trials evaluating personalized treatment algorithms based on CD138 and NK cell status could serve as the next crucial step toward individualized reproductive care [15].

An additional frontier of exploration involves the use of non-invasive diagnostic tools to evaluate endometrial immune status. Recent studies have investigated the potential of uterine fluid aspiration (“endometrial flushing”) as a less invasive alternative to biopsy for detecting immune markers. Pilot data suggest that soluble forms of CD138 and NK-associated cytokines (e.g., IL-15, IFN-γ) can be quantified in endometrial secretions and may correlate with intra-tissue findings [15].

This technique could enable repeated monitoring of immune profiles over time or in response to treatment, which is particularly useful in ART protocols. However, standardization of collection methods, assay validation, and correlation with histological data remain necessary steps before clinical implementation [19,35].

Such innovations may facilitate broader application of immune profiling, offering valuable insights while minimizing procedural discomfort and risk for patients undergoing fertility evaluation [19,35].

The interaction between chronic inflammation (CD138^+^) and innate immune hyperactivity (NK cells) may reflect a shared dysregulation at the metabolomic level [35]. Preliminary findings suggest altered levels of metabolites involved in oxidative stress and immune cell metabolism (e.g., glutamine, tryptophan–kynurenine pathway), which may affect local cytokine production and immune tolerance. Such changes may modulate NK cytotoxic thresholds and plasma cell survival, further impairing implantation. Integration of transcriptomic and proteomic signatures into multivariate models could eventually support composite immune diagnostics in clinical settings [19,35].

This integrated model supports the idea of “immune-mediated infertility,” in which multiple immune components synergistically (or antagonistically) influence reproductive success [19,35]. In ART settings, CD138^+^ plasma cells and NK cell abnormalities have shown particular relevance in frozen embryo transfer (FET) cycles under hormone replacement therapy (HRT), where the lack of endogenous immunomodulation by the corpus luteum may increase susceptibility to immune dysfunction. For instance, studies have shown that antibiotic treatment of chronic endometritis prior to IVF-FET significantly improved clinical pregnancy and live birth rates [32,37]. Similarly, in women with elevated peripheral NK cytotoxicity undergoing ICSI, the use of corticosteroids or intralipids prior to embryo transfer has been associated with increased implantation rates [34]. These findings suggest that immunological markers such as CD138 and NK cell activity may help guide pre-treatment decisions in ART candidates, particularly those with repeated implantation failure or unexplained infertility. An integrated immunological evaluation may also support individualized embryo transfer timing and the use of adjunctive therapies, such as corticosteroids, IVIG, or antibiotics, depending on the patient’s immune profile.

As endometrial immune profiling becomes increasingly complex, the integration of artificial intelligence (AI) and machine learning (ML) offers promising tools for interpreting multidimensional data. Preliminary studies have applied AI algorithms to datasets containing CD138^+^ cell counts, NK cell activity, cytokine profiles, and clinical variables (e.g., age, endometrial thickness, prior ART cycles) to predict implantation outcomes with encouraging accuracy. Such approaches may help identify patient subgroups that could benefit most from immunomodulatory therapy or altered ART timing. While these models remain in early development, they underscore the potential of computational tools in personalizing fertility care and enhancing clinical decision-making.

While the integration of artificial intelligence, machine learning, and metabolomics into reproductive immunology presents exciting opportunities, it is important to note that these approaches are still in early exploratory phases. To date, only a limited number of empirical studies have applied AI algorithms to predict implantation outcomes using immunological and clinical data, and these studies often involve small cohorts or pilot datasets. Similarly, research on metabolomic profiling of the endometrial immune environment is nascent, with preliminary findings suggesting possible associations between metabolic dysregulation and immune cell activity but lacking large-scale validation. As such, these tools should currently be viewed as promising adjuncts rather than established components of clinical practice. Future studies with robust designs and larger sample sizes are necessary to confirm their diagnostic and prognostic utility in the context of infertility.

### 4.4. Strengths and Limitations of the Literature

Unlike previous reviews that have focused on either CD138^+^ plasma cells or NK cells in isolation, this review presents an integrated perspective on how chronic endometrial inflammation and innate immune dysregulation may jointly compromise reproductive outcomes. By examining their interaction, we propose a composite immunological framework that may guide more nuanced diagnostic strategies and support the use of sequential or combined immunomodulatory therapies. This dual-marker approach represents an emerging paradigm in the investigation of idiopathic infertility, offering a more tailored and potentially effective clinical pathway.

Strengths:

- Growing number of studies examining endometrial immunology in infertility [8,20,24].
- Use of specific techniques (e.g., immunohistochemistry, flow cytometry) for cell identification [7,13,17].
- Initial evidence supporting the effectiveness of targeted therapies (e.g., antibiotics, immunotherapy) in improving reproductive outcomes in patients with immune abnormalities [32,34].

Limitations:

- High methodological heterogeneity across included studies [17,31].
- Lack of large-scale randomized controlled trials (RCTs) [38].
- Absence of standardized cut-offs for interpreting immune markers [26,31].
- Limited exploration of interactions between immune cell types and underlying molecular mechanisms [19,37].

Another significant limitation of the current literature is the underrepresentation of male factor infertility in studies examining immune-mediated infertility. While this review focuses on endometrial immunity, the interplay between paternal antigens, seminal fluid, and maternal immune modulation remains poorly understood. Seminal plasma contains immunomodulatory factors that influence the uterine environment post-coitus. An altered seminal profile—due to infection, inflammation, or dysbiosis—might shape maternal immunity in a way that predisposes to CE or abnormal NK cell activation.

Furthermore, racial and ethnic disparities in immune marker expression are rarely addressed. Genetic polymorphisms in KIR and HLA-C genes vary across populations, which may influence the generalizability of findings. Most included studies are conducted in East Asian or European populations, limiting extrapolation to diverse clinical contexts.

Finally, patient heterogeneity—including different ART protocols, stimulation regimens, embryo stages, and biopsy timing—complicates interpretation and comparison of outcomes. Future studies should aim for harmonization of clinical protocols and incorporate multi-omics approaches (e.g., transcriptomics, microbiomics, proteomics) to clarify molecular underpinnings.

In summary, this review supports a significant association between endometrial immune alterations and couple infertility [8,10,14]. However, translating these findings into clinical practice will require greater methodological rigor, diagnostic standardization, and validation in future studies [37,38,39,40,41].

In addition to the biological and clinical implications, the psychological impact of immunological infertility diagnostics warrants careful consideration. For many couples, receiving a diagnosis involving chronic endometritis or abnormal NK cell activity can provide a sense of relief, replacing uncertainty with a tangible explanation and pathway for treatment. However, such diagnoses may also provoke anxiety, particularly when the therapeutic evidence is still evolving or when treatment involves prolonged or off-label immunomodulatory strategies. Unclear prognostic value or ambiguous immune profiles (e.g., borderline CD138^+^ counts or fluctuating NK cell activity) can further compound emotional distress [39,40,41]. Studies in reproductive psychology have shown that diagnostic labeling in infertility—especially when it involves the immune system—can alter patients’ self-perception and expectations toward ART outcomes. As such, the introduction of immune profiling into fertility care should be paired with psychological counseling and transparent communication regarding diagnostic uncertainty, therapeutic options, and realistic outcome expectations. Addressing this psychosocial dimension is essential to ensure that the benefits of personalized medicine do not inadvertently increase emotional burden [40,41,42].

A significant limitation highlighted by our review is the absence of unified reference values for both CD138^+^ plasma cells and uterine NK cells. Cutoff thresholds, diagnostic methods (e.g., immunohistochemistry vs. flow cytometry), and biopsy timing vary considerably across studies, leading to substantial methodological heterogeneity. This variability hampers direct comparisons and limits the ability to draw firm conclusions or develop standardized clinical recommendations. Until consensus is reached on diagnostic criteria and quantification techniques, the interpretation and clinical applicability of these immune biomarkers will remain constrained.

Another methodological limitation of this review is the exclusive reliance on the PubMed/MEDLINE database. While PubMed provides broad access to peer-reviewed biomedical literature, the omission of additional databases such as Scopus, Web of Science, and Embase may have led to selection bias or exclusion of relevant studies not indexed in PubMed. We also acknowledge that grey literature, including conference abstracts and unpublished studies, was not explored, potentially limiting comprehensiveness.

Furthermore, although our search and selection strategy was informed by PRISMA principles, we did not conduct a formal risk-of-bias assessment using tools such as the Cochrane Risk of Bias tool or the Newcastle–Ottawa Scale. This choice reflects the narrative design of the review, which aimed to generate a broad, integrative synthesis rather than a quantitative meta-analysis. The lack of standardized outcome measures, diagnostic thresholds (e.g., for CD138^+^, CD56, and BCL-6 expression), and methodological heterogeneity among included studies further limits the feasibility of formal quality grading.

Additionally, we recognize substantial heterogeneity in the definition, detection methods, and clinical interpretation of immunological biomarkers. Diagnostic thresholds for CD138^+^ plasma cells vary from ≥1 to ≥5 cells per high-power field, while NK cell quantification methods differ across studies (e.g., flow cytometry vs. immunohistochemistry). BCL-6, proposed as a marker of endometrial progesterone resistance, also lacks standardized reference ranges, complicating comparisons.

Despite these limitations, emerging data support the potential value of a composite immune panel—including CD138^+^, CD56, and BCL-6—as the foundation for an “immune-mediated infertility” model. This integrated approach may help identify women with subtle but clinically relevant endometrial immune dysregulation, particularly in cases of unexplained infertility, recurrent implantation failure (RIF), or recurrent pregnancy loss. Such panels, when applied within well-characterized clinical protocols, could facilitate individualized ART decision-making—e.g., determining the timing of embryo transfer, the need for endometrial immunomodulation (e.g., antibiotics, corticosteroids, or IVIG), or the appropriateness of frozen vs. fresh cycles.

However, until robust prospective studies validate the predictive value and cost-effectiveness of these panels, their use should remain investigational. Future systematic reviews on this topic should prioritize the use of multi-database search strategies, incorporate formal quality appraisal, and aim to stratify results by assay type, immune marker thresholds, and patient phenotype. This will be essential to transition from exploratory immune profiling to clinically actionable reproductive immunology.

### 4.5. Psychological Considerations and Counseling Needs

An important but often underappreciated limitation in the clinical implementation of immunological infertility diagnostics is the psychological burden they may impose on patients. Ambiguous or borderline findings—such as equivocal CD138^+^ plasma cell counts, variable NK cell profiles, or inconclusive BCL-6 expression—can lead to increased anxiety, confusion about prognosis, and emotional distress. For many patients, the absence of standardized thresholds or validated treatment pathways may undermine confidence in the diagnosis and raise concerns about overtreatment or uncertain benefit. To mitigate these effects, we recommend that immune profiling be accompanied by structured psychological counseling, particularly in individuals with a history of unsuccessful ART or unexplained infertility. Transparent communication about the evolving nature of immune testing, its limitations, and the current level of evidence is essential to preserve informed decision-making and patient well-being.

### 4.6. Future Perspectives

Despite significant advances, key challenges remain regarding diagnostic standardization, patient selection, and study quality. Future research should promote prospective, multicenter randomized studies to validate the routine clinical use of these immune markers and to develop shared interpretation guidelines [40,41,42]. Incorporating endometrial immunology into reproductive medicine requires close collaboration between gynecologists, immunologists, pathologists, and reproductive endocrinologists. Only through multidisciplinary efforts and well-designed prospective studies can we establish validated protocols that translate immune knowledge into improved fertility outcomes. Identifying and modulating endometrial immune profiles through analysis of CD138^+^ plasma cells and NK cells represents a promising frontier in infertility management. A multidisciplinary approach involving gynecologists, immunologists, and fertility specialists will be essential to improve diagnostic accuracy and therapeutic efficacy for infertile couples. Future directions should include the development of clinical algorithms that incorporate immune biomarkers alongside hormonal, anatomical, and genetic assessments. The cost-effectiveness and practicality of integrating such diagnostics into routine fertility care remain to be validated, but initial models suggest potential benefits in reducing ART failure rates [40,41,42].

Additionally, patient selection is crucial. Immunological assessments may be most useful in those with idiopathic infertility, repeated IVF failure, or unexplained miscarriage. Expanding our understanding of immunological fertility profiles in these subgroups may allow for more nuanced risk stratification and targeted interventions.

The integration of endometrial immune biomarkers into clinical infertility practice could benefit from adopting a tiered diagnostic strategy. In such an approach, initial screening using hysteroscopy or ultrasound could identify structural anomalies, followed by targeted immunohistochemical evaluation for CD138^+^ cells and NK markers in patients with unexplained infertility, RIF, or RSA.

Moreover, combining immunological data with clinical and biochemical markers—such as progesterone levels, uterine artery Doppler flow, and anti-endometrial antibodies—could offer a more holistic assessment of endometrial receptivity. In ART settings, such stratification may help personalize embryo transfer timing or identify candidates for immune-modulating therapies [40,41,42].

Pharmacologic modulation of uterine immunity remains an area of active research. While antibiotics are standard for CE, immunosuppressive strategies (e.g., corticosteroids, tacrolimus) and immunostimulatory agents (e.g., G-CSF) are being explored for NK-related dysfunction. The challenge lies in selecting the appropriate intervention based on a reliable, reproducible immune profile.

Artificial intelligence and machine learning models also hold promise in interpreting complex immunological data and predicting implantation outcomes. Preliminary algorithms using combined clinical and immunohistochemical inputs have shown reasonable predictive power for ART success. However, such tools require robust external validation.

From a public health perspective, widespread immune screening in infertility patients raises questions of cost-effectiveness, especially in resource-limited settings. Efforts should focus on defining high-yield populations for testing, such as those with multiple failed ART cycles, recurrent loss, or long-standing idiopathic infertility.

Another consideration is the psychological impact of immune-based diagnoses on infertile couples. The recognition of an “immunological cause” for infertility can provide relief and validation but also introduce anxiety regarding treatment complexity and prognosis. Therefore, counseling should accompany diagnostic disclosure, helping patients navigate therapeutic options with realistic expectations.

As immune diagnostics become more sophisticated, clinicians must also be cautious of over-medicalization. Not every immune abnormality warrants intervention, and clear thresholds for defining pathological vs. physiological immune states are urgently needed to avoid overtreatment [40,41,42].

Finally, policy frameworks and insurance systems must adapt to support these evolving diagnostic models. Funding for immunological assessments, and reimbursement for tailored therapies, will be essential to avoid inequalities in access and to truly integrate personalized medicine into reproductive care.

Together, these considerations highlight that translating immune research into clinical benefit requires not just scientific validation but also thoughtful ethical, psychological, and policy-level integration [40,41,42].

## 5. Conclusions

Couple infertility is a multifactorial clinical condition in which the immune system, though often overlooked in routine care, is increasingly recognized as a key player in regulating the endometrial environment and embryo implantation. This narrative review shows that CD138^+^ plasma cells and Natural Killer cells—particularly their uterine subtype (uNK)—play significant roles in mechanisms that may hinder conception and pregnancy maintenance.

The integrated assessment of multiple endometrial immune markers—such as CD138, CD56, and BCL-6—holds promise for a more complete immunological profile and for a personalized diagnostic approach. This combined strategy may underpin a more precise, individualized reproductive medicine model.

This narrative review highlights that both CD138^+^ plasma cells and uterine Natural Killer cells play significant and potentially synergistic roles in the pathophysiology of couple infertility, particularly in cases of unexplained infertility, recurrent implantation failure (RIF), and recurrent miscarriage. CD138^+^ plasma cells are strongly associated with chronic endometritis, a treatable condition that, when diagnosed through immunohistochemistry, can lead to improved ART outcomes following targeted antibiotic therapy.

Similarly, while NK cells are essential for a healthy endometrial environment, excessive numbers or cytotoxic activity—especially in the absence of clear diagnostic thresholds—have been linked to impaired implantation and pregnancy maintenance. Our synthesis underscores the potential benefit of a combined immunological assessment of CD138^+^ and NK cells, which may provide a more comprehensive diagnostic and therapeutic framework for managing idiopathic infertility.

Future clinical protocols should prioritize the development of standardized diagnostic criteria, clarify therapeutic indications, and integrate psychosocial support. These efforts are essential to translate immunological findings into actionable tools that improve reproductive outcomes.

The novelty of this review lies in its emphasis on the interplay between CD138^+^ plasma cells and uterine NK cells, advocating for a combined immunological assessment in women with unexplained infertility or repeated ART failure. This integrated approach may allow for improved diagnostic precision and personalized treatment, setting the stage for future research and the development of standardized clinical protocols.

In conclusion, the immune landscape of the endometrium—particularly the interplay between CD138^+^ plasma cells and NK cells—represents a compelling domain for innovation in reproductive medicine. Ongoing advances in diagnostics, therapeutics, and technology are poised to transform this emerging field from exploratory to actionable in routine clinical care. Multidisciplinary collaboration and patient-centered approaches will be pivotal in harnessing this potential for improving fertility outcomes.

## Data Availability

No new data were created or analyzed in this study. Data sharing is not applicable to this article.

## References

[1] Zegers-Hochschild F., Adamson G.D., de Mouzon J., Ishihara O., Mansour R., Nygren K., Sullivan E., van der Poel S., International Committee for Monitoring Assisted Reproductive Technology, World Health Organization (2009). International Committee for Monitoring Assisted Reproductive Technology (ICMART) and the World Health Organization (WHO) revised glossary of ART terminology, 2009. Fertil. Steril..

[2] Mascarenhas M.N., Flaxman S.R., Boerma T., Vanderpoel S., Stevens G.A. (2012). National, regional, and global trends in infertility prevalence since 1990: A systematic analysis of 277 health surveys. PLoS Med..

[3] Cousineau T.M., Domar A.D. (2007). Psychological impact of infertility. Best Pract Res Clin Obstet Gynaecol..

[4] Thoma M.E., McLain A.C., Louis J.F., King R.B., Trumble A.C., Sundaram R., Louis G.M.B. (2013). Prevalence of infertility in the United States as estimated by the current duration approach and a traditional constructed approach. Fertil. Steril..

[5] Cissen M., Bensdorp A.J., Cohlen B.J., Repping S., de Bruin J.P., van Wely M. (2016). Assisted reproductive technologies for male subfertility. Cochrane Database Syst Rev..

[6] Robertson S.A. (2005). Seminal plasma and male factor signalling in the female reproductive tract. Cell Tissue Res..

[7] Mor G., Cardenas I. (2010). The immune system in pregnancy: A unique complexity. Am. J. Reprod. Immunol..

[8] Nancy P., Tagliani E., Tay C.-S., Asp P., Levy D.E., Erlebacher A. (2012). Chemokine gene silencing in decidual stromal cells limits T cell access to the maternal-fetal interface. Science.

[9] Lédée N., Petitbarat M., Rahmati M., Chaouat G., Dubanchet S. (2021). New pre-conception immune biomarkers for clinical practice: Interleukin-15 and interleukin-18 binding protein. J. Reprod Immunol..

[10] Kitaya K., Yasuo T. (2010). Aberrant expression of cell adhesion molecules in the endometrium of infertile patients with chronic endometritis. Mod. Pathol..

[11] Cicinelli E., Resta L., Nicoletti R., Zito A., Piccoli R., Marziani G. (2005). Detection of chronic endometritis at fluid hysteroscopy. J. Minim. Invasive Gynecol..

[12] Johnston-MacAnanny E.B., Hartnett J., Engmann L.L., Nulsen J.C., Sanders M.M., Benadiva C.A. (2010). Chronic endometritis is a frequent finding in women with recurrent implantation failure after in vitro fertilization. Fertil. Steril..

[13] Bulmer J.N., Morrison L., Longfellow M., Ritson A., Pace D. (1991). Granulated lymphocytes in human endometrium: Histochemical and immunohistochemical studies. Hum. Reprod..

[14] Quenby S., Kalumbi C., Bates M., Farquharson R., Vince G. (2005). Prednisolone reduces preconceptual endometrial natural killer cells in women with recurrent miscarriage. Fertil. Steril..

[15] Kitaya K., Nagai Y., Arai W., Sakuraba Y., Ishikawa T. (2019). Characterization of Microbiota in Endometrial Fluid and Vaginal Secretions in Infertile Women with Repeated Implantation Failure. Mediators Inflamm..

[16] Tang A.W., Alfirevic Z., Turner M.A., Drury J.A., Li T.C., Quenby S. (2013). A feasibility trial of screening women with idiopathic recurrent miscarriage for high uterine natural killer cell density and randomizing to prednisolone or placebo when pregnant. Hum. Reprod..

[17] Seshadri S., Sunkara S.K. (2014). Natural killer cells in female infertility and recurrent miscarriage: A systematic review and meta-analysis. Hum. Reprod. Update.

[18] Iwasawa T., Takahashi T., Maeda E., Ishiyama K., Takahashi S., Suganuma R., Matsuo K., Tachibana M., Fukuhara R., Shirasawa H. (2021). Effects of localisation of uterine adenomyosis on outcome of in vitro fertilisation/intracytoplasmic sperm injection fresh and frozen-thawed embryo transfer cycles: A multicentre retrospective cohort study. Reprod. Biol. Endocrinol..

[19] Liu Y., Chen X., Huang J., Wang C.-C., Yu M.-Y., Laird S.M., Li T.-C. (2018). Comparison of the prevalence of chronic endometritis as determined by means of different diagnostic methods in women with and without reproductive failure. Fertil. Steril..

[20] Changaei M., Javidan M., Ramezani Tehrani F., Mosaffa N., Noroozzadeh M., Hosseinzadeh R., Rajaei S. (2023). Reduced expression of Il10, Stat3, Hoxa10, and Itgb3 in the embryo implantation site of rat model with prenatal androgen-induced polycystic ovary syndrome. Am. J. Reprod. Immunol..

[21] Page M.J., McKenzie J.E., Bossuyt P.M., Boutron I., Hoffmann T.C., Mulrow C.D., Shamseer L., Tetzlaff J.M., Akl E., Brennan S.E. (2021). The PRISMA 2020 statement: An updated guideline for reporting systematic reviews. BMJ.

[22] Falagas M.E., Pitsouni E.I., Malietzis G.A., Pappas G. (2008). Comparison of PubMed, Scopus, Web of Science, and Google Scholar: Strengths and weaknesses. FASEB J..

[23] Warriner D. (2008). How to Read a Paper: The Basics of Evidence-Based Medicine. BMJ..

[24] Kitaya K., Tada Y., Hayashi T., Taguchi S., Funabiki M., Nakamura Y. (2014). Comprehensive endometrial immunoglobulin subclass analysis in infertile women suffering from repeated implantation failure with or without chronic endometritis. Am. J. Reprod. Immunol..

[25] Moher D., Liberati A., Tetzlaff J., Altman D.G., PRISMA Group (2009). Preferred reporting items for systematic reviews and meta-analyses: The PRISMA statement. PLoS Med..

[26] Higgins J.P.T., Thomas J., Chandler J., Cumpston M., Li T., Page M.J., Welch V.A. (2022). Cochrane Handbook for Systematic Reviews of Interventions, Version 6.3.

[27] Wells G.A., Shea B., O’Connell D., Peterson J., Welch V., Losos M., Tugwell P. (2012). The Newcastle-Ottawa Scale (NOS) for Assessing the Quality of Nonrandomised Studies in Meta-Analyses.

[28] Higgins J.P.T., Savović J., Page M.J., Sterne J.A.C. (2022). Assessing risk of bias in a systematic review. Cochrane Handbook for Systematic Reviews of Interventions, Version 6.3.

[29] Shang J., Wang S., Wang A., Li F., Zhang J., Wang J., Lv R., Chen H., Mu X., Zhang K. (2024). Intra-ovarian inflammatory states and their associations with embryo quality in normal-BMI PCOS patients undergoing IVF treatment. Reprod. Biol. Endocrinol..

[30] Kasius J.C., Broekmans F.J., Sie-Go D.M., Bourgain C., Eijkemans M.J., Fauser B.C., Devroey P., Fatemim H.M. (2012). The reliability of the histological diagnosis of endometritis in asymptomatic IVF cases. Hum. Reprod..

[31] McQueen D.B., Bernardi L.A., Stephenson M.D. (2015). Chronic endometritis in women with recurrent early pregnancy loss and/or implantation failure: A study of diagnostic variability. Fertil. Steril..

[32] Bouet P.E., El Hachem H., Monceau E., Gariépy G., Kadoch I.J., Sylvestre C. (2016). Chronic endometritis in women with recurrent pregnancy loss and recurrent implantation failure: Prevalence and role of office hysteroscopy and immunohistochemistry in diagnosis. Fertil. Steril..

[33] Fukui A., Fujii S., Yamaguchi E., Kimura H., Sato S., Saito Y. (1999). Natural killer cell subpopulations and cytotoxicity for infertile patients undergoing in vitro fertilization. Am. J. Reprod. Immunol..

[34] Coulam C.B., Acacio B. (2012). Does immunotherapy for treatment of reproductive failure enhance live births?. Am. J. Reprod. Immunol..

[35] Lédée N., Petitbarat M., Chevrier L., Vitoux D., Vezmar K., Rahmati M., Dubanchet S., Gahéry H., Bensussan A., Chaouat G. (2016). The Uterine Immune Profile May Help Women With Repeated Unexplained Embryo Implantation Failure After In Vitro Fertilization. Am. J. Reprod. Immunol..

[36] Lédée N., Petitbarat M., Prat-Ellenberg L., Dray G., Cassuto G.N., Chevrier L., Kazhalawi A., Vezmar K., Chaouat G. (2020). Endometrial Immune Profiling: A Method to Design Personalized Care in Assisted Reproductive Medicine. Front. Immunol..

[37] Deshpande P.S., Gupta A.S. (2019). Causes and Prevalence of Factors Causing Infertility in a Public Health Facility. J. Hum. Reprod. Sci..

[38] Pandian Z., Gibreel A., Bhattacharya S. (2012). In vitro fertilisation for unexplained subfertility. Cochrane Database Syst. Rev..

[39] Libretti A., Savasta F., Nicosia A., Corsini C., De Pedrini A., Leo L., Laganà A.S., Troìa L., Dellino M., Tinelli R. (2024). Exploring the Father’s Role in Determining Neonatal Birth Weight: A Narrative Review. Medicina.

[40] Dumancic S., Bakotin Jakovac M., Mimica M.D., Zekic Tomas S., Marusic J. (2025). CD56-Positive NK Cells and CD138-Positive Plasma Cells in Basal Decidua of Term Placentas in Singleton Pregnancies after Assisted Reproductive Technology Treatment of Endometriosis-Related Infertility. Life.

[41] Chiokadze M., Bär C., Pastuschek J., Dons’koi B.V., Khazhylenko K.G., Schleußner E., Markert U.R., Favaro R.R. (2020). Beyond Uterine Natural Killer Cell Numbers in Unexplained Recurrent Pregnancy Loss: Combined Analysis of CD45, CD56, CD16, CD57, and CD138. Diagnostics.

[42] Dwojak E., Mroczek M., Dworacki G., Dobosz P., Ślubowska A., Stępień M., Borowczyk M., Filipczyńska I., Tomaszewska A., Ałtyn R. (2024). Plasma Cells as the Key Players of IVF Failure? Unlocking the Enigma of Infertility and In Vitro Fertilization Failure in the Light of Uterine Inflammation. Int. J. Mol. Sci..

